# Quantification of Interdependent Dynamics during Laser Additive Manufacturing Using X‐Ray Imaging Informed Multi‐Physics and Multiphase Simulation

**DOI:** 10.1002/advs.202203546

**Published:** 2022-10-31

**Authors:** Chu Lun Alex Leung, Dawid Luczyniec, Enyu Guo, Sebastian Marussi, Robert C. Atwood, Martina Meisnar, Ben Saunders, Peter D. Lee

**Affiliations:** ^1^ Department of Mechanical Engineering University College London Torrington Place London WC1E 7JE UK; ^2^ Research Complex at Harwell Science & Technology Facilities Council Rutherford Appleton Laboratory Oxfordshire OX11 0QX UK; ^3^ Rolls Royce plc Elton Road Site, North Block Derby DE24 8BJ UK; ^4^ Key Laboratory of Solidification Control and Digital Preparation Technology (Liaoning Province) School of Materials Science and Engineering Dalian University of Technology Dalian 116024 China; ^5^ Diamond Light Source Ltd Harwell Science & Innovation Campus Oxfordshire OX11 0DE UK; ^6^ European Space Agency ESA‐RAL Advanced Manufacturing Laboratory Harwell‐Oxford Campus Fermi Avenue Didcot OX110FD UK

**Keywords:** additive manufacturing, imperfection, multiphase, pore, simulation, XCT, X‐ray imaging

## Abstract

Laser powder bed fusion (LPBF) can produce high‐value metallic components for many industries; however, its adoption for safety‐critical applications is hampered by the presence of imperfections. The interdependency between imperfections and processing parameters remains unclear. Here, the evolution of porosity and humps during LPBF using X‐ray and electron imaging, and a high‐fidelity multiphase process simulation, is quantified. The pore and keyhole formation mechanisms are driven by the mixing of high temperatures and high metal vapor concentrations in the keyhole is revealed. The irregular pores are formed via keyhole collapse, pore coalescence, and then pore entrapment by the solidification front. The mixing of the fast‐moving vapor plume and molten pool induces a Kelvin–Helmholtz instability at the melt track surface, forming humps. X‐ray imaging and a high‐fidelity model are used to quantify the pore evolution kinetics, pore size distribution, waviness, surface roughness, and melt volume under single layer conditions. This work provides insights on key criteria that govern the formation of imperfections in LPBF and suggest ways to improve process reliability.

## Graphical abstract

Revealing pore evolution and metal vaporization mechanisms during additive manufacturing of Ni‐alloys using X‐ray and electron imaging, and high‐fidelity simulation, including: a) pore characteristics; b) grain texture; and c) high‐speed X‐ray images showed pore movement, coalescence and growth during AM. The high‐fidelity simulation model was used to quantify the solid‐liquid‐argon‐vapor interactions on: d) pore evolution; and e) metal vaporization mechanisms.

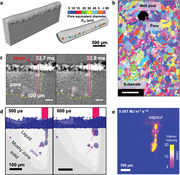

## 1 Introduction

Laser powder bed fusion (LPBF) is a form of additive manufacturing (AM) that transforms a 3D digital design into a net‐shaped component by selectively fusing powder particles with a focused laser beam, layer upon layer.^[^ ^1^ ^]^ AM is widely used in many industries, including the aerospace, automotive, biomedical, energy and naval sectors;^[^ ^2^, ^3^ ^]^ however, it is less frequently used for safety‐critical applications, for example, turbine blades or ship propellers, in demanding operating environments owing to potential process and product inconsistencies.^[^ ^4^, ^5^ ^]^ Some AM parts may exhibit microstructural anisotropy^[^ ^6^ ^]^ and imperfections,^[^ ^7^ ^]^ including pores,^[^ ^8^, ^9^ ^]^ micro‐cracks,^[^ ^10^, ^11^ ^]^ inclusions^[^ ^12^ ^]^ and high surface roughness,^[^ ^13^, ^14^ ^]^ leading to undesirable mechanical performance and resulting in premature failure during service.

Minimizing imperfections in AM components is of utmost interest to industry, as they affect process dynamics^[^ ^15^, ^16^ ^]^ and overall manufacturing productivity; therefore, a better understanding of the mechanisms by which imperfections occur during printing is essential to progress AM technology.^[^ ^17^ ^]^ Over the last decade, extensive work has been performed to investigate the formation of imperfections and multi‐phase interactions between the powder particles (solid), molten pool (liquid), shielding gas, and metallic plume (vapor) in LPBF using in situ monitoring techniques,^[^ ^18^, ^19^ ^]^ for example, high‐speed optical,^[^ ^20^, ^21^ ^]^ infra‐red^[^ ^19^, ^22^, ^23^ ^]^ and schlieren imaging.^[^ ^24^ ^]^ With the ultrafast laser–matter interaction time (10^−3^ to 10^−6^ s)^[^ ^25^ ^]^ and opaque melt pool surface, high‐speed X‐ray imaging has been used to reveal and elucidate the pore and melt pool dynamics during LPBF under overhang,^[^ ^8^, ^9^, ^26^ ^]^ keyhole regime,^[^ ^27^, ^28^ ^]^ laser turning,^[^ ^16^ ^]^ and multi‐layer conditions.^[^ ^26^, ^29^, ^30^ ^]^ These studies provided insights into different pore evolution mechanisms, including keyhole collapse,^[^ ^27^, ^28^ ^]^ pore coalescence, dissolution, precipitation,^[^ ^8^, ^9^ ^]^ vaporization of volatile elements,^[^ ^9^ ^]^ pore flow behavior driven by the centrifugal^[^ ^8^, ^15^, ^28^ ^]^ and centripetal^[^ ^26^ ^]^ Marangoni convection, and the formation of open pores.^[^ ^26^ ^]^ These in situ results focus on the influence of process parameters on spherical pores^[^ ^9^, ^15^, ^16^, ^31^, ^32^, ^33^ ^]^ and have less emphasis on the formation mechanisms of irregular pores,^[^ ^26^ ^]^ for example, lack of fusion.^[^ ^34^ ^]^ Three common empirical methods have been used to correlate the effects of process parameters on the resultant microstructures, including: 1) “power–velocity process design charts”;^[^ ^35^ ^]^ 2) “normalized model‐based process diagram”;^[^ ^36^ ^]^ 3) “normalized enthalpy method”;^[^ ^37^, ^38^ ^]^ and 4) “normalized enthalpy products.”^[^ ^39^ ^]^ These methods can be used to minimize pore formation in AM but they have less emphasis when it comes to predicting other imperfections, for example, surface topology. To devise and implement a printing strategy that minimizes different types of imperfections, we must better understand the interdependent relationship between the process parameters, multi‐phase interactions and formation mechanisms of imperfections.

Meso‐scale process simulation models have been used to address some of the missing physical understanding of how these imperfections are formed during AM at high spatial (µm) and temporal resolution (µs).^[^ ^40^, ^41^, ^42^, ^43^, ^44^ ^]^ Many of these models have not coupled the full multi‐phase interaction, mostly simulating only two‐phases, for example, liquid–solid or liquid–gas, rather than multi‐phases, to shorten the simulation time and to avoid hefty computational costs. However, it remains unclear which assumptions should be made to simplify these models. A key challenge to advance these models is the requirement for high spatiotemporal experimental data and temperature‐dependent thermophysical properties for model validation and verification. Therefore, our knowledge of how imperfections are formed and their relationship with processing parameters in LPBF remains incomplete, and improving this will be crucial for process and product qualification.

Here, we reveal the evolution of pores and humps during LPBF of Inconel 625 powder using in situ high‐speed X‐ray imaging and correlate these results with high‐resolution electron and X‐ray microscopy to quantify the surface and internal quality of AM components in 3D. All results are then compared to the imparted specific energy, SE = $\frac{P}{{\left(v.d\right)}^{0.5}}$ (MJ m^−1^ s^−1/2^)^[^ ^45^ ^]^ on the LPBF process where *P*, *v* and *d* are the laser power, scan velocity and laser beam diameter, respectively. The experimental data is used to verify a multi‐phase and multi‐physics process simulation. Our high‐fidelity 3D simulation model predicts temperature, melt pool geometry, fluid velocity, multi‐phase (solid–liquid–vapor) interaction at a spatial‐temporal resolution of 1.25 µm and 10 ns. The model accurately captures the highly dynamic and nonlinear laser–matter interactions between solid, liquid, vapor, and argon gas. It also incorporates temperature‐dependent thermophysical properties from 373 to 10 000 K with an adaptive laser heating factor. Using in situ X‐ray imaging and synchrotron calibrated high‐fidelity model, we have devised five simplified empirical formulae to quantify the interdependent relationship between the SE, pore kinetics, melt volume and build features, such as microstructural features (e.g., pores, waviness, and surface roughness), for the AM Inconel 625 tracks. These formulae can be applied to develop low fidelity process simulations to reduce computational resources. This work provides a foundation for designing next‐generation optimization routines for the production of AM tracks.

## 2 Results and Discussion

### 2.1 Evolution of Regular and Irregular Pores During LPBF

**Figure 1:**
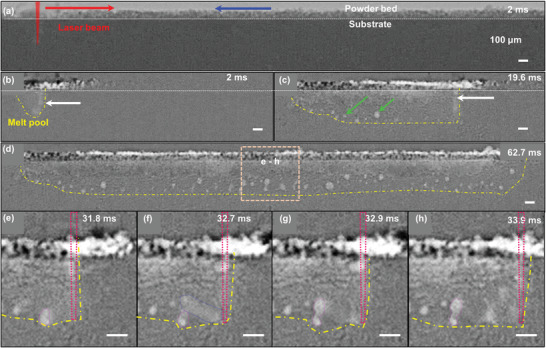
Time‐series radiographs show the formation of melt track and pores during LPBF of Inconel 625 (*P* = 150 W, *v* = 50 mm s^−1^, *t* = 100 µm, *d* = 50 µm, and SE = 0.095 MJ m^−1^ s^−1/2^). a) A radiograph before background subtraction (BGS) wherein light grey, medium grey and jet‐black colors show argon/background, Inconel 625 powder and substrate, respectively. The red arrow indicates the direction of the scanning laser beam, the blue arrow indicates the argon gas flow direction, and the yellow dotted line indicates the melt region. Selected BGS images reveal the pore formation phenomena at b) 2, c) 19.6, and d) 62.7 ms with a region of interest. The magnified images (e–h) show the formation of irregular pores where f) a gas pore is entrained by the Marangoni convection, g) merges with a pre‐existing pore, and h) retains its shape and position after solidification. Scale bar 100 µm. See details in Movie S1, Supporting Information.

**Figure** **1** shows time‐series radiographs during LPBF of Inconel 625 at a specific energy (SE) of 0.095 MJ m^−1^ s^−1/2^. Slow scan speed was used to match the layer thickness of 100 µm and the X‐ray imaging capabilities. Figure 1a is a typical radiograph captured during the in situ experiment. We enhanced the image contrast of the keyhole geometry, porosity, and melt zone by applying a background subtraction (BGS) technique on all radiographs (described in Experimental Section 4.1). At the onset of the LPBF process, the laser beam melts a layer of powder particles whilst slowly drilling into the substrate. The penetration depth increases steadily until it reaches a maximum value of ca. 450 µm when the laser beam moves 400 µm away from its starting position (see white arrows in Figure 1b,c and Video S1, Supporting Information).

Following the Kirchhoff's law of thermal radiation, some of the incoming laser energy is reflected and some of the laser energy is absorbed from the powder bed surface, transforms as heat, before conducting across the powder bed and the substrate. It takes time to form a vapor depression zone, and hence the penetration depth increases steadily. Once the vapor depression zone is formed, the highly focused laser beam (with a laser power density of ca. 10^7^ W cm^−2^) melts and vaporizes the free surface of the depression zone, inducing a recoil pressure that pushes the molten metal downward and sideways. Laser drilling deepens the depression zone as the number of laser reflections and laser absorbance increase within the interaction zone,^[^ ^46^ ^]^ forming a keyhole. Eventually, the keyhole reaches its maximum depth because the laser‐induced metal vapor^[^ ^24^ ^]^ or/and plasma at the bottom of the depression zone, and subsequent laser–vapor–plasma interaction weakens the incoming beam intensity via Fresnel or inverse Bremsstrahlung absorption.^[^ ^46^ ^]^

The complex multi‐phase interaction prevents further laser drilling (or increase in keyhole depth) as laser scanning continues because of the varying localized input laser energy at the free surface of the vapor depression zone, causing perturbations at the free surface of the keyhole and changing the keyhole shape over time. The keyhole morphology observed in this study is constantly transforming from a “J‐shaped” (at 2 ms) into an “I‐shaped” keyhole (at 19.6 ms), see Figure 1 b,c and Movie S1. In the reverse scanning direction, the J‐shaped would appear as reversed J‐shaped. This observation is different from prior work^[^ ^47^ ^]^ which reports a distinct keyhole morphology at a given scanning parameter. Though recent work reveals keyhole shape changes in welding scenarios,^[^ ^48^ ^]^ some suggested that the keyhole shapes during LPBF are linked to a combination of laser power,^[^ ^49^ ^]^ scan velocity^[^ ^47^ ^]^ and powder layer thickness.^[^ ^30^ ^]^ The local variations in powder packing density, absorptivity and thermal diffusivity of the powder bed can also alter the applied localized enthalpy, leading to inhomogeneous heating and melting during LPBF.^[^ ^49^ ^]^

As laser melting progresses, spherical pores continue to form at the root and trailing edge of the keyhole (Figure 1c and Movie S1, Supporting Information). The evolution of spherical pores matches well with prior hypotheses, for example, pores could initiate from the collapse of an unstable keyhole^[^ ^27^ ^]^ due to a combination of multiple‐beam reflection and Fresnel absorption at the vapor depression zone.^[^ ^25^ ^]^ On further cooling, we observed the pore movement and the fast‐flowing liquid metal are both driven by the Marangoni convection. As the melt pool cools, the formation of gas pores may arise from melting and solidification^[^ ^8^ ^]^ or entrapment of the shielding gas, for example, argon or nitrogen, inside the build chamber.

Figure 1d displays a final snapshot of the experiment wherein the region of interest reveals the formation of irregular pores in the melt track. This has not been previously observed and it is distinctively different from traditional hypotheses in the field. Prior work suggested that the formation of irregular pores is due to a lower energy input,^[^ ^7^ ^]^ arising from the gaps between partially melted powder particles or powder layers,^[^ ^50^ ^]^ or partial melting of a large pore from a previously built layer.^[^ ^26^ ^]^

These enlarged images (Figure 1e–h) and Movie S1 reveal that a keyhole pore (purple) is first entrained in the opposite direction of the scanning laser beam via the centrifugal Marangoni convection (Figure 1f). The motion blur (the blue dotted region in Figure 1f) demonstrates that the flow velocity of the pore (and liquid metal) is at least 1 m s^−1^ (as the pore moves ca. 200 µm backward over 0.2 ms). The estimated flow velocity is similar to that reported under overhang conditions^[^ ^8^ ^]^ which is ca. 1.6 and 0.45–4.5 m s^−1^ in a single layer melt track.^[^ ^15^, ^34^, ^51^ ^]^ After that, the keyhole pore (32.7 ms) coalesces with a pre‐existing pore (31.8 ms), forming an irregular pore with a peanut shape (Figure 1g,h). These peanut pores are repeatedly observed during LPBF as shown by the X‐ray computed tomography (XCT) result in Figure S1, Supporting Information. Upon solidification, the pore shape remains irregular as the surrounding material near this pre‐existing pore (31.8 ms) appears to be in a solid state. The temperature surrounding the pore remains high, allowing the mixture of metal vapor and other gas, for example argon, hydrogen, nitrogen, etc. to diffuse into the irregular pore from the (semi‐)solid, and increasing its internal gas pressure. By calculating the internal pressure of the irregular gas pore using a 3D rendered volume from XCT, the pressure force exerted from the pore surface (ca. 10^−3^ N) is sufficiently high to overcome a combination of the buoyancy force (ca. 10^−6^ N), drag force (ca. 10^−6^ N), and Marangoni‐driven force (ca. 10^−6^ N) imposed on the pore (see details in Supporting Information). The pressure exerted from the gas pore is also sufficiently strong to resist the deformation induced by grain growth and retain the pore shape during solidification. Figure 1d, and Movie S1, and Figure S1, Supporting Information, demonstrate that the melt track produced at high specific energy (0.095 MJ m^−1^ s^−1/2^) exhibits many irregular (peanut‐shaped) pores and their formation mechanism is driven by keyhole collapse, followed by pore coalescence, validating our prior hypotheses.^[^ ^30^ ^]^

**Figure 2:**
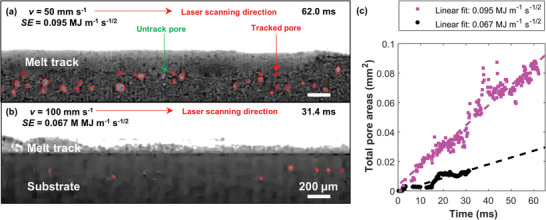
2D pore area quantification during LPBF of Inconel 625. Exemplary radiographs overlaid with tracked pores (red) at SE of a) 0.095 and b) 0.067 MJ m^−1^ s^−1/2^; c) pore area analysis on experiment (a) and (b); the line of best fit equations are: *y* = 1.359 × 10^−3^ *x* − 3.803 × 10^−3^ (*r* ^2^ = 0.829, *p*‐value = 0.0005) and *y* = 4.63 × 10^−4^ *x* − 4.999 × 10^−4^ (*r* ^2^ = 0.925, *p*‐value = 0.0014), respectively. Scale bar 200 µm. Pore tracking videos are available in Movies S2 and S3, Supporting Information.

To explain the kinetics of the pore evolution process in LPBF, we quantified the changes in pore area over time, that is, the pore area growth rate (**Figure** **2**), from two different specific energies, SE (or scan velocity, *v*). The location and size of the extracted pores (see red outlines) during LPBF are shown in Figure 2a,b and Movies S2 and S3, Supporting Information. The melt track produced by a high SE of 0.095 MJ m^−1^ s^−1/2^ exhibits many more irregular and keyhole pores (Figure 2a) than that produced by a low SE of 0.067 MJ m^−1^ s^−1/2^ (Figure 2b). The time‐resolved pore area analysis (Figure 2c) shows that the total pore areas (or pore formation process) increase linearly with time, and the pore growth rate equals the gradient of the regression line. The total pore area reduces by a factor of ca. five and the pore growth rate reduces by a factor of ca. three with decreasing SE from 0.095 to 0.067 MJ m^−1^ s^−1/2^. This empirical relationship demonstrates a possible pore reduction strategy by lowering SE or increasing *v* in LPBF, which complements the adaptive power map strategy proposed by ref. [16].

To further explain this empirical relationship, we employ our high‐fidelity multiphase model to elucidate the underlying physics behind the pore evolution mechanisms under three other experimental conditions and then cross‐validate these simulation results with XCT and SEM‐electron backscatter diffraction (EBSD).

### 2.2 Multiphase Interaction during LPBF

**Figure 3:**
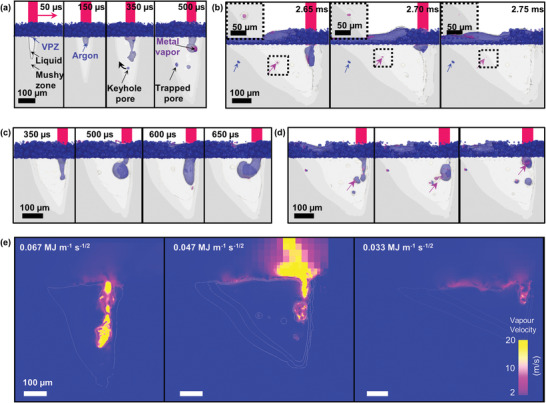
Multiphase simulation results showing melt pool and defect dynamics during LPBF of Inconel 625. At the onset of LPBF, a) laser drilling occurs and formation of keyhole pores at the vaporization depression zone (VPZ); b) pore shrinkage due to cooling and condensation of metal vapor, see pore evolution events (inset); c) the *I*–*J* keyhole transformation during LPBF where the keyhole cavity mainly contains argon gas; d) keyhole pore formation due to keyhole instability induced by mixing of metal vapor and argon gas; e) shows the reduction of porosity and keyhole depth with decreasing specific energy, *SE*. All scale bars are 100 µm. See details in Videos S4–S6, Supporting Information.

We used the synchrotron X‐ray images to calibrate a multi‐phase and multi‐physics simulation model (see details in Experimental Method) which predicts the melt pool and defect dynamics during LPBF. Our simulation results (**Figure** **3**a) show that the vapor depression zone forms ca. 50 µs after the laser switches on (SE = 0.067 MJ m^−1^ s^−1/2^), capturing the laser drilling process, and the subsequent pore formation caused by the keyhole instability (similar to Figure 1a). Before the keyhole reaches its maximum depth, some keyhole pores are formed and then trapped inside the melt track (500 µs) upon solidification due to insufficient time to float toward the melt pool surface and then burst into the atmosphere^[^ ^8^ ^]^ or restriction of pore movement due to the acoustic waves generated from the keyhole collapse.^[^ ^52^ ^]^

Unlike a typical LPBF simulation model, our multiphase simulation model can estimate different concentrations of argon gas (blue color) and/or metal vapor (pink color) inside the vapor depression zone (or keyhole) and voids as shown by Figures 1 and 3a–d. At the onset of LPBF, the vapor depression zone is mainly filled with argon gas (Figure 3a). As LPBF progresses, some pores are made up of a mixture of argon gas and metal vapor (Figure 3b). Those pores containing a high concentration of metal vapor are expected to shrink in pore volume by a factor of >2 (based on the ideal gas law) as the melt pool cools from 3500 to ∼1563 K (solidus temperature of Inconel625). The pore may also shrink as the metal vapor condensesat the pore/liquid interface. Upon the completion of solidification, these pores become vacuum voids (see insets of Figure 3b) while those containing insoluble argon gas are retained in the simulated track as gas pores. Once the pore is trapped in the solid, the pore size will be fixed, but the pressure inside the pore will reduce. This simulation model did not consider the bulk and surface chemistry of the powder; therefore, we cannot completely disregard other potential pore evolution mechanisms. These mechanisms include pore formation due to a gas release of hydrogen^[^ ^30^ ^]^ or nitrogen from the powder composition, argon or nitrogen from gas atomized powders^[^ ^50^ ^]^ (Supporting Information), or a release of CO_2_ from the decomposition of adventitious carbon at the powder surface during LPBF.^[^ ^26^, ^53^ ^]^

### 2.3 Cyclic I–J Shaped Keyhole Transformation

The high‐fidelity simulation model reproduces the I–J transformation throughout LPBF by taking into account the presence of multiphase interaction and only two laser reflections from the free surface of the vapor depression zone.^[^ ^54^ ^]^ With decreasing SE from 0.067 to 0.047 MJ m^−1^ s^−1/2^, a cyclic transformation between I‐ and J‐ shaped keyholes is evident at an interval of ca. 50–150 µs or a frequency of between 6 and 20 kHz (Figure 3c). The oscillation frequency in LPBF is similar to that reported in laser metal processing, however, the laser power density in our current study is 10 times higher than that reported in ref. [54] and their relationship remains unclear.

As the metal vaporization increases at the bottom of the keyhole, this induces a recoil pressure and creates a cavity in the melt pool, forming an I‐shaped keyhole. Due to further laser–matter interaction, the vapor plume expands at the interaction zone and is combined with the strong backward Marangoni flow and jet of the vapor plume generated by the laser reflections, causing the keyhole shape to elongate toward the back of the melt pool, forming a J‐shaped keyhole. The I–J keyhole transformation occurs in the sub‐millisecond range (Figure 3c) rather than 10 ms (Figure 1b) no pores are observed in either case due to the limitation of the imaging facility. The changes in keyhole shape have been reported in laser metal processing^[^ ^48^ ^]^ and LPBF^[^ ^29^ ^]^ using ultra‐fast X‐ray imaging and simulation;^[^ ^55^, ^56^ ^]^ however, none discussed the correlations between cyclic I‐J keyhole transformation and pore formation mechanisms.

### 2.4 Relationship between Keyhole Pores and Vapor Plume

The keyhole pores are less likely to form when the vapor depression zone is only filled with argon gas (Figure 3c). The keyhole becomes more unstable when it contains a mixture of argon gas and metal vapor (Figure 3d). The mixing of fast‐moving high‐temperature metal vapor and argon gas (up to 20 m s^−1^ and >5000 K) initiates the keyhole transition wherein the fast‐moving liquid metal is driven by the Marangoni convection and pinches the middle/top of the keyhole (Figure 3d), reducing the waist of the cavity. Additionally, the localized metal vapor inside and above the laser–matter interaction zone is partially shielding the laser beam and causing inhomogeneous heating at the melt surface, resulting in a perturbation at the keyhole free surface. This leads to keyhole collapse and the bottom of the keyhole pinches off to form pores. Prior in situ work has reported that pores do not always form during keyhole LPBF^[^ ^29^, ^57^ ^]^ and pore formation is commonly considered as a stochastic event.^[^ ^58^ ^]^ Our work reveals that the initial perturbation that leads to pore formation is complex and stochastic; however, the propensity of this stochastic event is deterministic. The keyhole pore formation is highly dependent on the metal vapor concentration within the keyhole cavity, that is, a high metal vapor concentration will lead to rigorous mixing, melt flow instability, keyhole collapse and subsequently pore formation.

**Figure 4:**
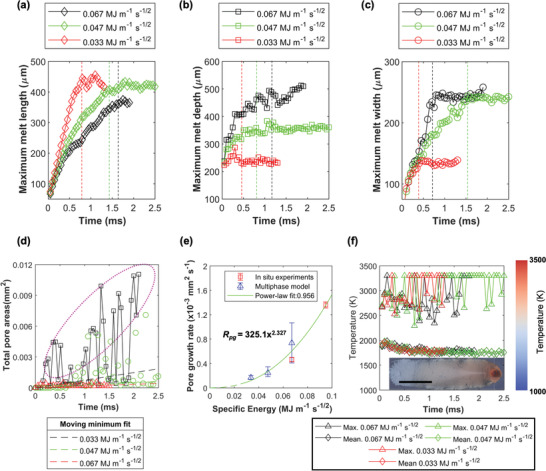
Quantification of melt pool geometry, pore growth rate, and surface temperature as a function of specific energy (SE) and time using high‐fidelity multiphase simulation. The a) length, b) depth, and c) width of the melt pool geometry are quantified over time where the vertical dotted lines in (a–c) show the time when the melt pool geometry reaches a plateau. d) The pore areas are quantified over time wherein the linear regression line is fitted based on moving minimum. The magenta circle highlighting a large number of pores is formed throughout the LPBF process; e) the pore growth rate results (*r* ^2^ = 0.956, *p*‐value = 0.00146), extracted from simulation (d) and experimental data (Figure 2c), and f) shows the maximum and average melt pool temperature where 3300 K is the maximum temperature of the liquid alloy; the scale bar is 250 µm.

**Figure 5:**
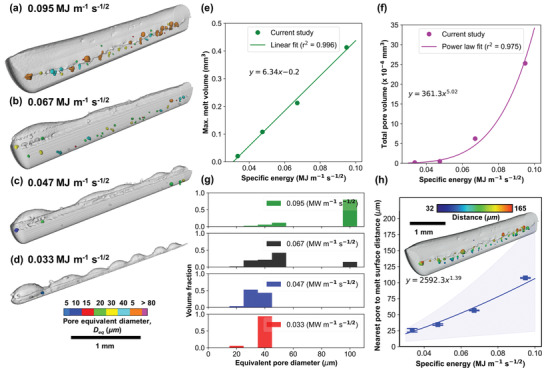
3D quantification of melt volume and pore volume analysis in Inconel 625 melt tracks as a function of specific energy (SE). 3D rendered melt track overlaid with internal pores at SE of a) 0.095, b) 0.067, c) 0.047, and d) 0.033 MJ m^−1^ s^−1/2^. The grey objects represent the melt volume, and the colored objects correspond to the pore volume equivalent diameter, *D*_eq_. Quantification results estimate the e) melt volume (*r* ^2^ = 0.996, *p*‐value = 0.0022), f) pore volume (*r* ^2^ = 0.975, *p*‐value = 0.00128), g) pore size distribution, and h) nearest pore‐to‐melt surface distance plot (*r* ^2^ = 0.975, *p*‐value = 0.00125). Plot (h) uses the standard error bar, and the blue shaded region covers the minimum and maximum measurements. The filled circle and square markers indicate the maximum and minimum nearest pore‐to‐melt surface distance, respectively. The inset shows melt track (a) is overlaid with pores with a colormap of the nearest pore‐to‐melt surface distance.

With decreasing SE, a shallower melt pool is formed during LPBF because this reduces both the laser–matter interaction time and absorbed energy at the vapor depression zone,^[^ ^56^ ^]^ minimizing the metal vaporization at the laser–matter interaction zone. The moving argon gas carries the metal vapor away from the melt zone due to the decrease in front wall angle^[^ ^47^ ^]^ or the decrease in recoil pressure due to the lower melt pool temperature.^[^ ^20^ ^]^ This reduces the mixing of metal vapor inside the keyhole and forms a stabilized vapor depression zone, lowering the probability of pore formation. If any pores are formed, they are usually trapped by the advancing solidification front (see Figures 3e, **4**, and **5**). From the melt track surface, the metal vapor cools as it moves along the track surface; altering the surface composition with a slight increase of the vaporized element(s).^[^ ^59^ ^]^ The simulation results emphasize that the melt pool shape and pore formation mechanisms during LPBF are governed by the metal vapor concentration within the keyhole cavity or vapor depression zone, and pore elimination would require good control of the metal vapor concentrations in LPBF.

### 2.5 Time‐Resolved Quantifications from the Multiphase Model

The simulated melt pool geometry only takes <2 ms (Figure 4a–c) to reach its maximum geometry. The results suggest that the time scale of the simulation is sufficient to capture the initial laser drilling event, keyhole instability, multiphase interaction, and the evolution of porosity and surface topology of AM tracks. Increasing the SE prolongs the laser–matter interaction time, resulting in smaller melt pools along the scanning direction whilst increasing its melt width and melt depth.

There is a huge fluctuation in the total pore areas over time (see the magenta circle in Figure 4d), up to 10 times more pores are formed during LPBF than those entrained in the simulated solidified track. This is due to a constant interaction between the laser beam and the pre‐existing pores (see quantification later), allowing gaseous species to float toward the melt surface and then burst, reincorporate or escape from the keyhole to the atmosphere, resulting in fewer pores in the AM track, which is consistent with our prior observations.^[^ ^8^, ^26^, ^30^ ^]^

Comparing the experimental (Figure 2c) with the simulation results (Figure 4d), a strong positive power law relationship (*r* ^2^ = 0.956) between the pore growth rate and SE (Figure 4e) is evident, the amount of porosity increases with reducing *v* or increasing SE. This empirical formula enables the prediction of pore evolution kinetics during LPBF of Inconel 625 without the need for intensive computational resources. We perform a separate analysis to describe the pore volume, morphology, distribution, etc. (see Figure 5). The derived formula can be extended to other powder materials using our proposed methods, further shortening the development of microstructural modelling and accelerating the materials development time for AM.

The high‐power density (>10^7^ W cm^−2^) laser beam is sufficient to cause metal vaporization across all the simulated conditions. The average and maximum liquid temperature on the melt track surface is calculated as ca. 1970 and 3200 K, respectively (Figure 4f). The maximum liquid temperature at the melt surface is bounded by the liquid–vapor transformation temperature; therefore, it remains at 3300 K. The volume of the metal vapor is expected to lower with decreasing SE^[^ ^56^ ^]^ and hence less vapor plume will be entrained in the molten pool (Figure 3e), reducing the final pore volume. This confirms that the volume of the metal vapor and its mixing behavior is a key mechanism that leads to the formation of keyhole pores, which has not been discussed or hypothesized in prior work.

### 2.6 Pore Elimination Mechanism by Reducing Specific Energy

These samples were further examined using XCT to study the influence of process parameters on the formation of porosity. Figure 5a–d display 3D rendered images of the melt track volume overlaid with its internal pores, corresponding to four different SE. They demonstrate that most pores are formed along the laser beam path (Figure S2, Supporting Information), consistent with prior work.^[^ ^60^ ^]^ Figure 5e shows a strong positive linear correlation between the melt volume and SE (*r* ^2^ = 0.996) as the higher SE provides more imparted energy for LPBF. The melt volume is estimated using SEM‐EBSD and XCT images (see Figure S4, Supporting Information). Increasing SE increases the melt depth and decreases the protrusion height of the melt track, because the liquid metal reaches over 1950 K (Figure 4f), which decreases its surface tension and allows it to spread instead of curling upward, also known as, balling. A strong positive exponential law relationship between the total pore volume and SE (*r* ^2^ = 0.975 shown in Figure 5f), confirming that a reduction of SE can minimise porosity in AM tracks (Figure 2c). The impact of SE on pore morphology and size distribution is shown in the pore fraction plots (Figure 5g). When SE ≥ 0.067 MJ m^−1^ s^−1/2^, large and irregular pores with a pore equivalent diameter, *D*_eq_, of ca. 100 µm are frequently formed. When the SE is reduced to 0.047 MJ m^−1^ s^−1/2^, this parameter eliminates large irregular pores but leads to the formation of spherical pores (see detailed pore analysis in Table S2, Supporting Information). Figure 5h measures the distance of the individual pore surface from its nearest melt pool surface. Fewer keyhole pores are being formed at the bottom of the melt pool at low SE (<0.047 MJ m^−1^ s^−1/2^) and their pore‐to‐melt surface distance is well below 50 µm. Compare with the track heights, these pores are mostly located in the middle of the melt track (supported by Figure 5c,d). This is because the Marangoni‐driven flow sweeps pores away from the depression zone and move them toward the top of the molten pool. Under the conditions studied, there is a strong correlation between SE, melt volume and pore volume, implying that these devised formulae can be used to optimize build rate (which corresponds to the melt volume) whilst reducing porosity to as low as reasonably practicable.

### 2.7 Influence of Specific Energy on Hump Formation

**Figure 6:**
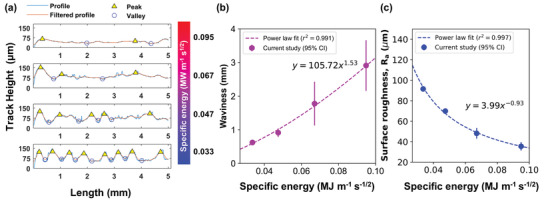
Topological analysis of Inconel 625 melt tracks as a function of specific energy (SE). a) The melt track profile, b) waviness (*r* ^2^ = 0.991, *p*‐value = 0.00465), and c) surface roughness (*r* ^2^ *=* 0.997, *p*‐value = 0.00167) plots as a function of SE.

An optimum parameter set for achieving the lowest porosity may not produce melt tracks with desired surface topology but a fine balance between different build qualities is paramount for the production of safety‐critical components. Under the conditions studied, a reduction of SE in LPBF could eliminate the formation of irregular pores; however, it can also lead to hump formation at the melt track surface (Figure 5). We used SEM and image processing to quantify the height maps, surface roughness, and waviness of the track profiles (**Figure** **6**). The results show that the topology of the samples changes from a smooth surface to a wavy surface, and then to rough surfaces with reducing SE. The waviness of the melt track decreases from 2.9 to 0.6 mm (Figure 6b) whilst the surface roughness (*R_a_*) along the length of the melt track (Figure 6c), increases from 35 to 91 µm. Our results also show that the hump formation follows a power–law correlation between SE and waviness or *R_a_* with a *r* ^2^ > 0.99.

These humps found in the low SE (≤ 0.047 MJ m^−1^ s^−1/2^) samples exhibit a similar protrusion height along the melt track whereas laser‐welded tracks usually have humps with disproportionate heights.^[^ ^61^ ^]^ There are several hypotheses on hump formation mechanisms in laser material processing,^[^ ^62^ ^]^ in which the hump formation in LPBF tracks is often linked to the Plateau–Rayleigh (P–R) instability.^[^ ^63^ ^]^ The P–R instability occurs when there is a pressure difference acting on the molten pool surface, causing the liquid stream to form humps.^[^ ^63^ ^]^ This instability is favored by a combination of: i) shallow molten pool with a high‐temperature dependent surface tension coefficient; and ii) small melt pool widths (*W*) and having a high melt pool length (*L*) to width ratio, such that the P–R instability is active when the condition *L* > 2*πW* is met.^[^ ^64^ ^]^ However, our simulation and experimental results show that the humping effect is not dominated by the Plateau–Rayleigh instability as *L <<* 2*πW* under all conditions studied, such that *L* can be up to 3 times shorter than 2*πW*, see details in Supporting Information.

Given that there are two fast‐flowing fluids (liquid metal and vapor plume) along the melt surface, this forms a stratification layer (at the gas–liquid interface). The differences between the velocities and densities of the fluids along the stratification layer can induce a perturbation along with the interface, forming humps or surface roughness, this is known as the Kelvin–Helmholtz (K–H) instability. Our high‐fidelity simulation results suggest that the hump formation in LPBF is linked to the K–H instability at the gas–liquid interface. We show that the gas–liquid interface becomes unstable and the K–H instability is active given that the following conditions are met: 1) *v*_g_ ≠ *v*_l_; and 2) ${\left(v_{g}-v_{l}\right)}^{2}>\frac{g(\rho_{g}^{2}-\rho_{l}^{2})}{k\rho_{g}\rho_{l}}$ (see Table S5, Supporting Information), where *v*_g_ and *v*_l_ are the average flow velocity of the vapor plume and the liquid metal; *ρ*_l_ and *ρ*_g_ are the density of the liquid metal and vapor plume, respectively; *g* is gravity and *k* is the inverse of the perturbation wavelength or waviness (*λ*).

Here, we can confirm that the K–H instability is active when SE *<* 0.067 MJ m^−1^ s^−1/2^ and it plays a more dominant role than P–R instability^[^ ^65^, ^66^ ^]^ in the hump formation mechanism during LPBF. Since the K–H instability is active, Wei's approximation^[^ ^67^ ^]^ $\lambda =\frac{\gamma }{\rho_{l}*{\left(v_{l}-v_{g}\right)}^{2}}$ could be used to estimate the waviness of these humps. This equation considers a combination of the K–H instability criterion and the surface tension force, *γ*, acting upon the gas–liquid interface. The *λ* is estimated as 0.2, 6.4 and 102 mm for the *SE* of 0.067, 0.047 and 0.033 MJ m^−1^ s^−1/2^, respectively. The *λ* increases with decreasing *SE*, showing an opposite trend than that observed by the experimental results. A possible explanation is that Wei's approximation assumes that the gas flow has a dominant effect on the gas–liquid interface, exerting a force equals to that exerted from the interface. Assuming *v*_l_ is ca. 2 m s^−1^, we estimated the gas velocities, *v*_g_, using the experimental waviness results, the thermophysical properties of Inconel 625 (Table S5, Supporting Information), and the goal‐seek function (MS office, USA). We found that the *v*_g_ at the interface is less than the mean *v*_g_ values (or the bulk gas flow) quantified by our model. This opposes the assumption of Wei's approximation of equally dominant forces from ambient gas flow and Marangoni‐driven flow, and hence future work is needed to adapt Wei's approximation to predict *λ*.

### 2.8 Microstructural Analysis

**Figure 7:**
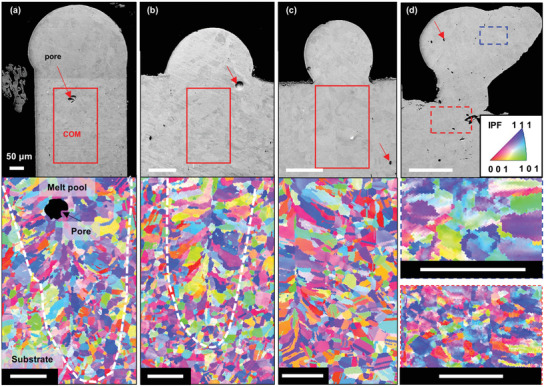
Backscattered electron images (BSEI) of Inconel 625 melt tracks processed at SE of a) 0.095, b) 0.067, c) 0.047, and d) 0.033 MJ m^−1^ s^−1/2^. The red or blue region of interest indicates the corresponding crystal orientation map (COM) with an inverse pole figure (IPF) key. The white dotted lines show the interface between the melt pool and the substrate. Scale bar 50 µm.

To further understand the process–structure–property relationship, we also examined the microstructure of these melt tracks using backscattered SEM, EBSD, and XRD analysis (**Figure** **7** and Figure S7, Supporting Information). The EBSD images (Figure 7a,b) show two distinctive microstructures within those samples produced at high SE: 1) fine equiaxed grains in the (hot‐rolled and annealed) Inconel 625 substrates; and 2) columnar grains in the melt track along the build direction and along the scanning direction, similar to what has been observed in a prior study.^[^ ^68^ ^]^ Unlike prior work,^[^ ^68^ ^]^ our samples show no texture as they did not undergo laser remelting. Figure 7c,d shows that the grain size reduces, and the grain morphology changes from columnar to columnar‐equiaxed transition and equiaxed as SE reduces from 0.067 to 0.047 and 0.033 MJ m^−1^ s^−1/2^, respectively. From these high‐resolution SEM images, we can observe that there are many pores having a *D*_eq_ of <5 µm within the melt track (Figure 7d). These pores have neither been detected nor quantified by in situ X‐ray radiography and XCT experiments due to the limited spatial resolution of the imaging setup. These pores are mainly spherical‐shaped, suggesting that they are gas pores which formed during solidification.^[^ ^26^ ^]^ The high‐resolution SEM image (Figure S5, Supporting Information) shows many fine precipitates of M_6_C, NbC, or *δ* ^[^ ^69^ ^]^ (<200 nm in size) in the *γ* matrix; however, they were not detected by XRD due to their low volume fraction (<5 vol%).

## 3 Conclusion

In summary, we uncovered and quantified the evolution mechanisms of the vapor depression (or keyhole), pores and hump during LPBF of Inconel 625 using X‐ray and electron imaging as well as a multi‐phase and multi‐physics simulation model. Our simulation results show that the keyhole collapse is mainly driven by the mixing of argon and metal vapor plumes, partially shielding the laser beam and causing inhomogeneous heating, resulting in keyhole pores. The keyhole pore formation is highly dependent on the metal vapor concentration within the keyhole cavity. Further, our experiments reveal the formation mechanism of irregular pores is mainly driven by keyhole collapse followed by pore coalescence with pre‐existing pores. We demonstrate that irregular pores can be mitigated by increasing scan velocity or reduction of specific energy, SE = $\frac{P}{{\left(v.d\right)}^{0.5}}$; however, this increases the surface roughness and waviness of the built track. Under the experimental conditions, we reveal that Kelvin–Helmholtz instability is a key perturbation that leads to hump formation, increasing the surface roughness of the melt track. Using these results, we derived five simple formulae to correlate SE with different process responses which are crucial for the production of LPBF parts, including build volume, pore volume, pore kinetics, surface roughness and waviness: Waviness (mm) = 105.72 *x* ^1.53^; surface roughness (µm) = 3.99 *x* ^−0.93^; Maximum melt volume (mm^3^) = 6.34 *x* − 0.2; pore volume (10^−4^ mm^3^) = 361.3*x* ^5.02^; and pore growth rate (10^−3^ mm^2^ s^−1^) = 325.1*x* ^2.327^ where *x* is SE. With increasing SE, we can improve the surface quality by minimising the waviness whilst lowering the surface roughness; however, this increases the melt volume, pore volume and formation of irregular pores, significantly altering the microstructure from columnar dendritic to equiaxed, vice versa. Here, we recommend using a low to medium SE to process Inconel 625 as it minimizes the formation of irregular pores and minimizes the surface roughness or waviness of the track surface. In future work, new studies will be required to determine the tunable coefficients (i.e., exponents) for these equations as the process responses of AM materials are governed by their thermophysical properties that could vary significantly from one system to the others.

## 4 Experimental Section

#### In Situ and Operando LPBF with Synchrotron X‐Ray Imaging

The in situ study explored the effects of laser scan velocity, *v*, and specific energy, SE = $\frac{P}{{\left(v.d\right)}^{0.5}}$ on the thermal fluid and defect dynamics during LPBF of Inconel 625. Here, an In Situ and Operando Process Replicator (ISOPR)^[^ ^8^, ^9^, ^26^ ^]^ was used to manufacture multiple single layer tracks while monitoring the LPBF process by operando high‐speed synchrotron X‐ray imaging at the beamline I12: Joint Engineering, Environmental, and Processing (JEEP), Diamond Light Source, UK.^[^ ^70^ ^]^

Cold‐rolled and annealed Inconel 625 foils (Special Metals Corporation, USA) were laser cut to a substrate dimension of 30 mm × 9.9 mm × 0.3 mm (length height × thickness). Each substrate was sandwiched by two boron nitride plates with a dimension of 30 mm × 100 mm × 0.3 mm (length × height × thickness), creating a powder bed with a fixed layer thickness of ca. 100 µm. A thick powder layer was used to match the small laser beam diameter (ca. 50 µm D4*σ_x_*_,_ *_y_*) in ISOPR. The powder bed assembly was held securely in a sample holder and then inserted into an environmental chamber. The experimental setup is outlined in ref. [9]. The laser power, *P*, was fixed at 150 W and *v* was set in between 100 and 400 mm s^−1^ *_._* For each run, a 5 mm track was produced using ISOPR in a flowing argon atmosphere at 4 l min^−1^.

All in situ X‐ray imaging experiments were captured by synchrotron X‐ray radiography at 5100 frames per second (fps) using 55 keV monochromatic X‐rays, custom module optics with a 700 µm thick LuAg: Ce scintillator coupled with a Miro 310M camera (Vision Research, USA). The field of view (FOV) of the camera was at 8.4 (width) and 3.3 mm (height) with a 6.6 µm pixel size. The image acquisition process is depicted in the authors’ previous work.^[^ ^8^, ^26^ ^]^ The acquired synchrotron radiographs were post‐processed and analyzed by MATLAB 2018a (MathWorks, USA). They were first normalized by flat‐field correction^[^ ^71^ ^]^ to remove image artefacts, followed by denoising using a video block‐matching (VBM3D) algorithm^[^ ^72^ ^]^ for noise reduction while preserving features of interest, for example, pores.^[^ ^8^ ^]^ Each denoised image was subjected to a background subtraction (BGS) that divides each denoised image by an average image derived from the denoised image stack. Triangle thresholding^[^ ^73^ ^]^ was used to binarize these BGS images and then quantified the pore area over time. The pore area analysis only considers objects with >5 pixels or having a pore area equivalent diameter (*D*_eq, *a*_) > 35 µm based on the current imaging setup. With *v* > 100 mm s^−1^, most pores having a *D*_eq,*a*_ < 35 µm do not appear in the radiographs, and hence two processing conditions were selected where *v* = 50 and 100 mm s^−1^ for such analysis.

#### Melt Track Analysis by X‐Ray Computed Tomography

After the in situ experiments, all samples were non‐destructively examined by XCT (Nikon XTH 225 X‐ray microfocus tomography system, Nikon, Japan). Each XCT scan was performed at 105 kV and 84 µA, comprising 2000 radiographic projections with a 1 s exposure time per projection. All scans were reconstructed into individual image volumes with a voxel size of 2.7 × 2.7 × 2.7 µm^3^ using built‐in beam hardening correction and filtered back projection algorithms embedded in CT Pro3D (Nikon, UK). All scans were post‐processed and quantified by Avizo 9.0 (Thermo Fisher Scientific, USA). First, they were subjected to a histogram stretching (or “Match Contrast”) operation to reduce image segmentation errors. This operation transformed all image volumes to have a histogram with a similar range of greyscale values. After that, these image volumes were subjected to a delineate filter with a kernel 3 × 3 × 3 voxels, followed by a 3D median filter with a kernel of 3 × 3 × 3 voxels for noise removal. The filtered image volume was segmented by the Otsu threshold,^[^ ^74^ ^]^ resulting in a segmented melt track on its substrate (Figure S4a, Supporting Information). The pore volume and pore sphericity were quantified using a standard pore analysis routine depicted in ref. [32].

The track height (*H*) and track width (*W*) from the XCT images was also measured using Avizo, the maximum melt depth (*D*) was verified by the X‐ray radiographs and SEM images, and used these measurements to generate a CAD model as a .stl file for each processing condition (Figure S4b, Supporting Information). Each .stl file was converted into an image volume (or a Mask image), and then registered to a corresponding binary image volume of the additive manufactured track (or a reference image), see Figure S4c, Supporting Information. The reference image was multiplied by the Mask, resulting in a 3D melt track image (Figure S4d, Supporting Information). After that, the volume of individual melt tracks was quantified using Avizo. Next, the pore (red) and melt track volume (alpha grey) were converted into triangular surfaces (Figure S4e, Supporting Information). Lastly, the distance between pore surfaces to the nearest surface of the extracted melt volume (e.g., Figure S4b, Supporting Information) were calculated, which revealed individual pore locations with respect to the melt volume and processing condition.

#### Waviness and Surface Roughness Analysis

The track profile was extracted using MATLAB 2019b. First, binarization was applied on the high‐resolution SEM images, followed by a maximum operation. The track profile was post‐processed using a moving mean window of 50. After that, the surface roughness was extracted by calculating the mean modulus of the smoothed track profile. Peak analysis was performed to extract the waviness by setting a minimum peak height of the surface roughness and a minimum peak prominence of the standard deviation of the smooth track profile.

#### Microstructural Analysis by Scanning Electron Microscopy

After XCT examinations, samples were sectioned in the middle and hot mounted in Bakelite (MulitFast resin, Struers). They were ground using 2000 and 4000 grit SiC papers, polished with diamond suspensions of 3 and 1 µm (Dia‐Duo‐2, Struers), and 0.01 µm colloidal silica suspension (OP‐S, Struers). After that, they were examined by backscattered electron imaging and EBSD orientation mapping using a Zeiss Leo 1530 VP SEM, equipped with an EDAX Hikari EBSD camera. These EBSD maps were acquired at an accelerating voltage of 20 kV and a step size of 0.5 µm. The grain size and grain misorientation were measured using EDAX OIM software and subsequently labelled with the inverse pole figure coloring.

#### Multi‐Phase Computational Fluid Dynamics for LPBF

Here, a finite volume method was used to simulate and predict the formation of keyhole, melt pool, and porosity during LPBF under similar experimental conditions as described in Section 4.1. This model takes into account solid–liquid–gas (Ar, vapor) interactions, laser–solid and laser–liquid interactions, phase transformations, melt pool behavior, gas–liquid boundary behavior, and temperature‐dependent thermophysical properties from 373 to 10 000 K. The simulations were carried out using existing solvers within the OpenFOAM Computational Fluid Dynamics framework^[^ ^75^ ^]^ and the powder was initialized in a pre‐sintered state to prevent powder motion and isolate the effects of spatter, similar to that reported in refs. [49, 76]. The multiphase, isothermal, incompressible, and volume‐of‐fluid (VOF) algorithms were adapted to capture changes of physical and thermodynamic effects in the multiphase system based on the available chemical and thermophysical data in refs. [77, 78].

First, the momentum, continuity, volume fraction, and energy equations were modified in the *“*icoReactingmultiphaseInterFoam.C*”* solver (OpenFOAM) according to refs. [79, 80, 81]:

(1) $$\frac{\partial \left(\rho u\right)}{\partial t}+\nabla \cdot \left(\rho \mathrm{uu}\right)=-\nabla p+\nabla \cdot \mu \left(\nabla u+\nabla u^{T}\right)+\mathrm{pg}+F_{\sigma }+F_{R}+D$$

(2) ∇·u=0

(3) $$\frac{\partial \alpha }{\partial t}+\nabla \cdot \left(\alpha u\right)+\nabla \cdot \left(\left(1-\alpha \right)\alpha \left(u_{1}-u_{2}\right)\right)=0$$

(4) $$\frac{\partial \left(\rho c_{p}T\right)}{\partial t}+u\cdot \nabla \left(\rho c_{p}T\right)-\nabla \cdot \left(\lambda \nabla T\right)=q_{L}+q_{H}$$

where *t* = time interval, *ρ* = density, *c*_p_ = specific heat capacity, *p* = pressure, *μ* = dynamic viscosity, *g* = gravitational acceleration, *u* = flow velocity, *T* = temperature, *λ* = thermal conductivity, *F*_*σ*_ = the surface tension force term, *F*_R_ = recoil force term, *q*_L_ = laser energy input, *q*_H_ = convective heat transport term, and *u*_1_ and *u*_2_ represent the velocity of phases 1 and 2 (for each of the phase pairs), respectively.

The volume fraction of each phase, *α*, in the system is defined by the following relationship:

(5) $$\alpha =\left\{\begin{array}{c} 0\\ 0<\alpha <1\\ 1 \end{array}\right.$$

OpenFOAM uses a specific implementation of conservation law for volume fraction, which is described by the surface compression scheme. The artificial compression term ∇ · ((1 − *α*)*α*(*u*_1_ − *u*_2_)) with compression velocity (*u*_1_ − *u*_2_) is zero within fluid domains where *α* = [0, 1]. It was used to reduce the interfacial width in a thin interfacial region.^[^ ^82^ ^]^

Since the VOF method is a pseudo one‐fluid approach, the following equations were added to calculate the local thermophysical properties of *ρ*, *μ*, and *λ*:

(6) $$\rho =\theta \rho_{1}+\left(1-\theta \right)\rho_{2}$$

(7) $$\mu =\theta \mu_{1}+\left(1-\theta \right)\mu_{2}$$

(8) $$\lambda =\theta \lambda_{1}+\left(1-\theta \right)\lambda_{2}$$

The phase fraction *α* is used for the phase fraction *θ* to track the interfaces between the solid–gas and solid–liquid phases.

The difficulty of the accurate numerical simulation with the VOF model (especially the keyhole behavior) lays within the approximation of surface tension force term *F*_*σ*_, as well as the tracking and reconstruction of the interface over time, *t*.^[^ ^83^ ^]^ In this high‐fidelity model, the Bénard–Marangoni convection was described with the modified Continuum Surface Force method and adapted the interfacial tangential continuum surface force component, *f*_*σ*,*τ*_, using a temperature‐dependent surface tension coefficient.^[^ ^81^, ^84^, ^85^, ^86^ ^]^

(9) $$\begin{array}{c} F_{\sigma }=f_{\sigma }\delta \left(\varphi \right)=\left(f_{\sigma ,n}+f_{\sigma ,\tau }\right)\delta \left(\varphi \right)=\left(\gamma \kappa n+\left(\gamma_{T}\left(\nabla T-n\left(n\cdot \nabla T\right)\right)\right)\right)\delta \left(\varphi \right) \end{array}$$

where *F*_*σ*_ = volumetric continuum surface force, *f*_*σ*_ = the surface force per unit area, *δ*(*φ*) = the delta function (accounting for the interface's thickness), *f*_*σ*,n_ = the normal component of the surface force, *κ* is the curvature of the interface, *κ* = − (∇ · n(*φ*)), $\gamma_{T}=-\left(\frac{\partial \gamma_{0}}{\partial T}\right)$ is the temperature‐dependent surface tension, ∇*T* is the temperature gradient, and $n=\frac{\nabla \Phi }{\left|\nabla \Phi \right|}$.

Besides using OpenFOAM multiphase solvers for mass and heat transfer, the vaporization force was further implemented acting at the liquid–vapor interface. This vaporization force was calculated based on the Hertz–Knudsen and Clausius–Clapeyron equations. The first formulation describes evaporation and condensation mass fluxes, and the second equation aids the quantification of the changes in pressure and temperature at saturation conditions. This yields the following equation to describe the mass transfer at the liquid–vapor interface (Open FOAM):

(10) $${\dot{m}}_{\mathrm{mR}}=\frac{2C}{2-C}\sqrt{\frac{M}{2\pi RT_{S}^{3}}}L_{\mathrm{vap}}\frac{\rho_{l}\left(T\right)\rho_{v}\left(T\right)}{\rho_{l}\left(T\right)-\rho_{v}\left(T\right)}\left(T-T_{s}\right)$$

where ${\dot{m}}_{\mathrm{mR}}$ = vapor mass flux rate, *C* = accommodation coefficient, *L* = latent heat of vaporization, *M* = averaged molecular weight of assumed vapor content (Cr, Ti, Al), R = gas constant, *T*_s_ = saturation temperature, $\rho_{v}^{T}$ = temperature‐dependent vapor density, and $\rho_{l}^{T}$ the temperature‐dependent liquid density.

The following equations were used to account for the recoil pressure and recoil force:^[^ ^87^ ^]^

(11) $$p_{\mathrm{sat},T}=p_{\mathrm{atm}}e^{\left\{-\frac{L_{\mathrm{vap},T_{0}}m_{\mathrm{mR}}}{k_{B}}\left[\frac{1}{T}\sqrt{1-{\left(\frac{T}{T_{\mathrm{crit}}}\right)}^{2}}-\frac{1}{T_{\mathrm{boil}}}\sqrt{1-{\left(\frac{T_{\mathrm{boil}}}{T_{\mathrm{crit}}}\right)}^{2}}-\frac{1}{T_{\mathrm{crit}}}\left(arcsin\left(\frac{T}{T_{\mathrm{crit}}}\right)-arcsin\left(\frac{T_{\mathrm{boil}}}{T_{\mathrm{crit}}}\right)\right)\right]\right\}}$$

(12) $$p_{\mathrm{recoil}}=p_{\mathrm{sat},T}\left(\frac{1+\beta_{R}}{2}\right)$$

(13) $$F_{R}=p_{\mathrm{recoil}}\left(\alpha_{2}n\nabla \alpha_{1}-\alpha_{1}n\nabla \alpha_{2}\right)$$

where *p*_sat,*T*_, saturation pressure at a given temperature, *α*_1_, *α*_2_ are the phase fractions of the liquid and vapor phases, respectively, *p*_atm_ = standard atmosphere, $L_{\mathrm{vap},T_{0}}$ = latent heat of vaporization at absolute zero, *k*_B_ = Boltzmann constant, *β*_R_ = recoil coefficient (assumed $\frac{p_{\mathrm{recoil}}}{p\mathrm{sat}}=0.56$ ^[^ ^87^ ^]^ for monatomic gases at the exit plane from the Knudsen layer at *Ma*_Kn_ = 1.0), *p*_recoil_ = recoil pressure, *T*_boil_ = boiling temperature at standard atmosphere (assumed *T*_boil_ = *T*_s_ = 3100 K), *T*_crit_, critical temperature = 3300 K at standard atmosphere, and *F*_R_ = the recoil force.

The level set approximation^[^ ^81^ ^]^ was carried out in several steps to improve the representation of the phase interfaces. First, the level set function, *φ*_0_, was initialized for the *α* field in the VOF model:

(14) $$\varphi_{0}=\left(2\alpha -1\right)\Gamma$$

where Γ = 0.75Δ*x* and Δ*x* is the cell size. The initial value is a signed distance function in which a positive value indicates the liquid phase, and a negative value indicates the gas phase.

The *φ*_0_ is then re‐distanced by solving the re‐initialization equation:

(15) $$\frac{\partial \varphi }{\partial \tau }=\mathrm{Sign}\left(\varphi_{0}\right)\left(1-\left|\nabla \varphi \right|\right)$$

where *φ*_0_ = *φ* (*x*, 0) and *τ* = 0.1Δ*x* are the initial level set function and the artificial time step, respectively, as shown by the *δ*(*φ*) Dirac function, in Equation (9):

(16) $$\delta \left(\varphi \right)=\left\{\begin{array}{cc} 0, & \left|\varphi \right|>\varepsilon \\ \frac{1}{2\varepsilon }\left(1+\cos\frac{\pi \varphi }{\varepsilon }\right), & \left|\varphi \right|\leq \varepsilon \end{array}\right.$$

where *ε* = *ε*_c_Δ*x* is an interface thickness with an arbitrary coefficient of the interfacial thickness *ε*_c_ determining the width of the *δ*(*φ*) Dirac function.

For the calculation of the local thermophysical properties of *ρ*, µ, and *λ* at the liquid–gas and liquid–vapor interfaces, the following Heaviside function was used, replacing *θ* phase fraction in Equations (6)–(8) by the level set formulation:

(17) $$H_{e}\left(\varphi \right)=\left\{\begin{array}{cc} 0, & \left|\varphi \right|<\varepsilon \\ \frac{1}{2}\left[1+\frac{\varphi }{\varepsilon }+\frac{1}{\pi }\sin\left(\frac{\pi \varphi }{\varepsilon }\right)\right], & \left|\varphi \right|\leq \varepsilon \\ 1, & \left|\varphi \right|>\varepsilon \end{array}\right.$$

To determine the phase transition, a simple enthalpy‐porosity formulation was used based on refs. [88, 89, 90]. The phase transformation was bound by the liquidus and solidus temperatures. Using these values, latent heat was update accordingly:

(18) $$\alpha_{L}=\left\{\begin{array}{cc} 0, & T<T_{S}\\ \frac{T-T_{S}}{T_{L}-T_{S}}, & T_{S}\leq T\leq T_{L}\\ 1, & T>T_{L} \end{array}\right.$$

(19) $$\Delta H=\alpha_{L}L_{m}$$

where *α*_L_ = fraction of the liquid, *T*_L_= liquidus temperature, *T*_S_ = solidus temperature,^[^^]^, Δ*H* = latent heat change, and *L*_m_ = latent heat of melting.

The momentum sink was introduced to limit the spurious currents in the mushy zone:

(20) $$D=-\frac{{\left(1-\alpha_{L}\right)}^{2}}{\left(\alpha_{L}^{2}+\delta \right)}C_{\mathrm{mush}}u$$

where *D* = momentum sink, *δ* = artificial coefficient prohibiting the division by zero, and the *C*_mush_ = momentum sink coefficient, which is in the range of 10^5^ to 10^11^_._^[^ ^92^ ^]^

The laser heat source was incorporated into the solver using the discrete ordinance method in OpenFOAM with ray‐tracing capabilities. The resultant heat transfer was governed by the laser radiation:

(21) $$q_{L}=a_{L}I_{0}=a_{L}\frac{2P_{L}}{{\pi \sigma }^{2}}e^{-\frac{2r^{2}}{\sigma^{2}}}$$

where *I*_0_ is the incoming laser beam intensity, *σ* is the standard deviation of the horizontal marginal beam distribution, *P*_L_ is the laser power, 150 W, *a*_L_ is the absorption coefficient, 0.327,^[^ ^93^, ^94^, ^95^ ^]^ and *r* is the beam radius at the focal position which is 25 µm in 4*σ*_*x*,*y*_ as shown in ref. [^8^].

#### Statistical Analysis

Image processing was carried out using MATLAB 2019b, ImageJ, and Avizo 2019.2. For statistical analysis, either MATLAB (MathWorks, Portola Valley, CA, USA), Python Scikit‐learn library (ver. 1.1.2)^[^ ^96^ ^]^ were used to calculate the mean, standard deviation, and the standard error values. For porosity analysis, up to 50 pores were tracked and quantified across the aforementioned specific energies.

## Conflict of Interest

The authors declare no conflict of interest.

## Author Contributions

C.L.A.L., B.S., and P.D.L. conceived the project. C.L.A.L. performs SEM, 2D radiography, topological characterization, XRD, and XCT analysis. E.G. performed XCT scanning. C.L.A.L., E.G., S.M., and R.C.A. set up and ran the in situ X‐ray imaging experiments. C.L.A.L., E.G., and M.M. prepared SEM track samples. M.M. performed SEM and EBSD. D.L. develops the multiphase and multiphysics simulation model and C.L.A.L. performs the simulation analysis. S.M. performs the gas flow simulation on the ISOPR. C.L.A.L. led the writing of the manuscript with all authors contributing.

## Supporting information

Supporting Information

Supplemental Video 1

Supplemental Video 2

Supplemental Video 3

Supplemental Video 4

Supplemental Video 5

Supplemental Video 6

## Data Availability

The data that support the findings of this study are available from the corresponding author upon reasonable request.
