# Supplementary material for: Quantification of Interdependent Dynamics during Laser Additive Manufacturing Using X‐Ray Imaging Informed Multi‐Physics and Multiphase Simulation
Source: Adv Sci (Weinh). 2022 Oct 31;9(36):2203546. doi: 10.1002/advs.202203546 (PMC9798986; doi:10.1002/advs.202203546)
Supplement: Supplementary file 1 — Supporting Information [file ADVS-9-2203546-s001.pdf]

Quantification of interdependent dynamics during laser additive manufacturing using X-ray imaging informed multi-physics and multiphase simulation

Chu Lun Alex Leung[1,2,*], Dawid Luczyniec[3], Enyu Guo[4], Sebastian Marussi[1,2], Robert C. Atwood[5], Martina Meisnar[6], Ben Saunders[3], Peter D. Lee[1,2,*]

[1] Department of Mechanical Engineering, University College London, Torrington Place, London WC1E 7JE, UK

[2] Research Complex at Harwell, Science & Technology Facilities Council, Rutherford Appleton Laboratory, Oxfordshire OX11 0QX, UK

[3] Rolls Royce plc., Elton Road Site, North Block, Derby, DE24 8BJ, UK

[4] Key Laboratory of Solidification Control and Digital Preparation Technology (Liaoning Province), School of Materials Science and Engineering, Dalian University of Technology, Dalian, China

[5] Diamond Light Source Ltd, Harwell Science & Innovation Campus, Oxfordshire, OX11 0DE, UK

[6] European Space Agency, ESA-RAL Advanced Manufacturing Laboratory, Harwell-Oxford Campus, Fermi Avenue, OX110FD Didcot, United Kingdom

[*] corresponding authors: alex.leung@ucl.ac.uk and peter.lee@ucl.ac.uk

1. Supplementary information

1.1. The pressure exerted in irregular pores

To support our hypothesis regarding the high-pressure gas pore (**Supplementary Figure 1**), the pressure force exerted from the pore F_{gas}, must equal to or exceed the sum of the applied forces $F_{applied}$, acting on the pore, including buoyant force (F_b), drag force(F_d), and Marangoni-driven force (F_m):

$$F_{gas} \geq F_{applied} \tag{1}$$

Firstly, we can assume that the pore is spherical, and it is insoluble in the liquid metal, the force exerted by the surface tension, F_{ST}, equals to the force exerted by the gas pressure, F_{gas} otherwise, the pore will shrink or grow.

$$F_{ST} = F_{gas} \tag{2}$$

$$F_{gas} = \frac{\rho RT}{M} * SA$$

Based on the ideal's gas law, P_{gas} increases proportional to the density, ρ, of the vapour plume, R is assumed to be the gas constant of 8.3145 J mol^{-1} k^{-1}, T is the temperature of the vapour plume, SA is the surface area of the pore, and M is the molecular mass of the vapour plume (argon + metal vapour) inside the pore. Although it is not possible to deduce ρ and M from our experiments, we know that F_{gas} must exceed the metallostatic pressure, P_{met}, of the thermal fluid exerted onto the pore surface. Given that P_{pore} >> P_{met}, we can use Laplace's law to estimate the metallostatic pressure:

$$P_{met} = \frac{\gamma(2\pi r_p)}{\pi r_p^2} = \frac{2\gamma}{r_p} \tag{3}$$

where γ is the gas-liquid interfacial energy (or surface tension) and r_p is the pore equivalent radius. Due to the lack of thermophysical data for Inconel 625, we assume the thermophysical properties of Inconel 625 are similar to Inconel 718. $\gamma_{Inconel718}$ is 1882 mN m^{-1} at 1609K [1], r_p is *ca.* 44 μm (taken from 3D pore analysis) and hence the P_{pore} is estimated to be 85.6 kPa or 0.85 atm. This is nearly 9 times the chamber pressure of 10 kPa.

$$P_{met} A_{surface} = F_{met} \tag{4}$$

where the pore surface area, $A_{surface}$ is 33685 μm^2, and hence F_{met} is calculated as 2.89 mN. We expect the F_{gas} is much greater than that of F_{met}.

The buoyant force (F_b) calculation:

$$F_b = \frac{4}{3}\pi r_p^3 \rho_l g \tag{5}$$

where r_p is the pore equivalent radius, ρ_l is the density of the liquid metal, and g is the gravitational acceleration, 9.8 m s^{-2}. Here, we also assume the density of Inconel 625 is similar to that of Inconel 718 where ρ_l = 7440 kg m^3 at 1609 K [1], and hence the F_b is estimated as 2.59 x 10^{-8} N.

The drag force (F_d) calculation:

$$F_d = -\frac{1}{2}C_D\rho_l\pi r_p^2\left(v_p - v_l\right)^2 \quad (6)$$

where C_D is the drag coefficient, ρ_l is the density of the liquid, r_p is the pore equivalent radius, v_p is the velocity of the pore, and v_l is the velocity of the liquid metal.

$$C_D = \frac{24}{Re}\,(1 + 0.15Re^{0.687}) \quad (7)$$

where Re is the Reynold number must be lower than 1000 and is given by:

$$Re = \frac{\rho_l\ |v_p - v_l|\ 2\ r_p}{\mu} = \frac{\rho_l * v_l * 2\ r_p}{\mu} \quad (8)$$

where ρ_l is the density of the liquid, r_p is the pore equivalent radius calculated from AVIZO, v_p is the velocity of the pore, v_l is the velocity of the liquid metal, and μ is the dynamic viscosity. To find the maximum drag force, we assume that F_d exerted on the pore with zero velocity, *i.e*, v_p is null, and therefore μ is ca. 0.009 Pa s (1638 K) [2] and v_l is *ca.* 1 m s^{-1} based on the radiography analysis. Re and C_D are estimated as 1.27 and 72.7, respectively. F_d is calculated as 6.46 x 10^{-7} N.

The Marangoni-driven force (F_m) calculation:

$$F_m = 4\pi r_p^2\left|\frac{\delta T}{\delta r}\right|\frac{\delta\sigma}{\delta T} \quad (9)$$

Where $\frac{\delta\sigma}{\delta T}$ is the temperature-dependent surface tension coefficient and $\frac{\delta T}{\delta r}$ is the temperature gradient at the pore location. For Inconel 718, $\frac{\delta\sigma}{\delta T} = -3.24 \text{ x } 10^{-4}\ \text{N m}^{-1}\text{K}^{-1}$and $\frac{\delta T}{\delta r} = 6.29 \text{ x } 10^{5}$ given that $\delta T = T_{liquidus} - T_{solidus} = 1638 - 1373 = 265K$ [2] and δr can be described as the melt depth and it is 421 µm (**Supplementary Figure 1**), therefore F_m is calculated as 4.95 x 10^{-6} N.

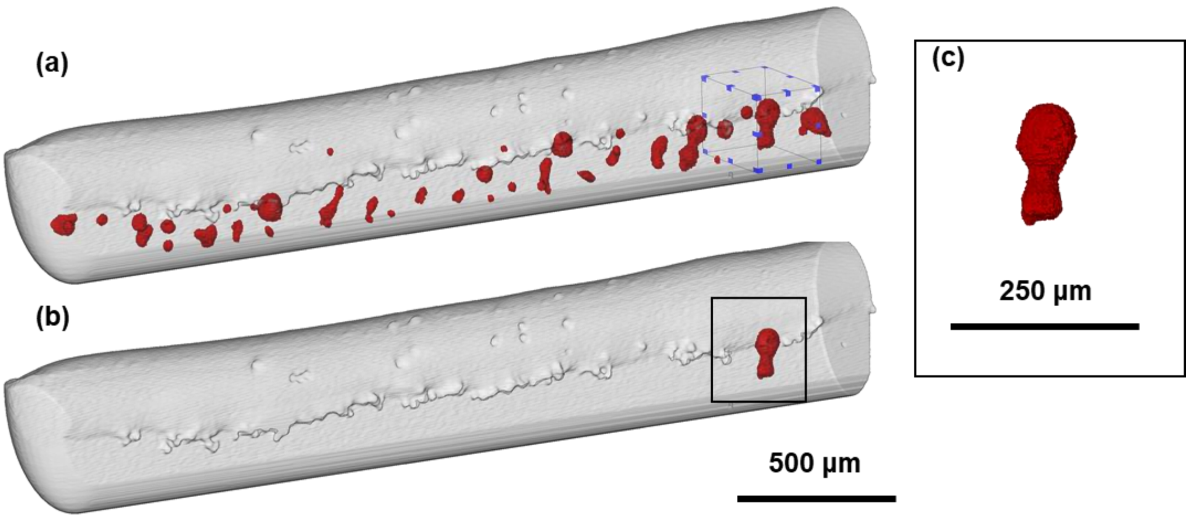

Supplementary Figure 1: XCT rendered images of a melt track at a specific energy (SE) of 0.095 MJ m^{-1} s$^{-1/2}$. **(a)** pores (red) overlaid to the melt volume wherein the region of interest is extracted by the cube. **(b)** illustrates the 'peanut' pore overlaid in the melt track and **(c)** a zoom-in image of the peanut pore.

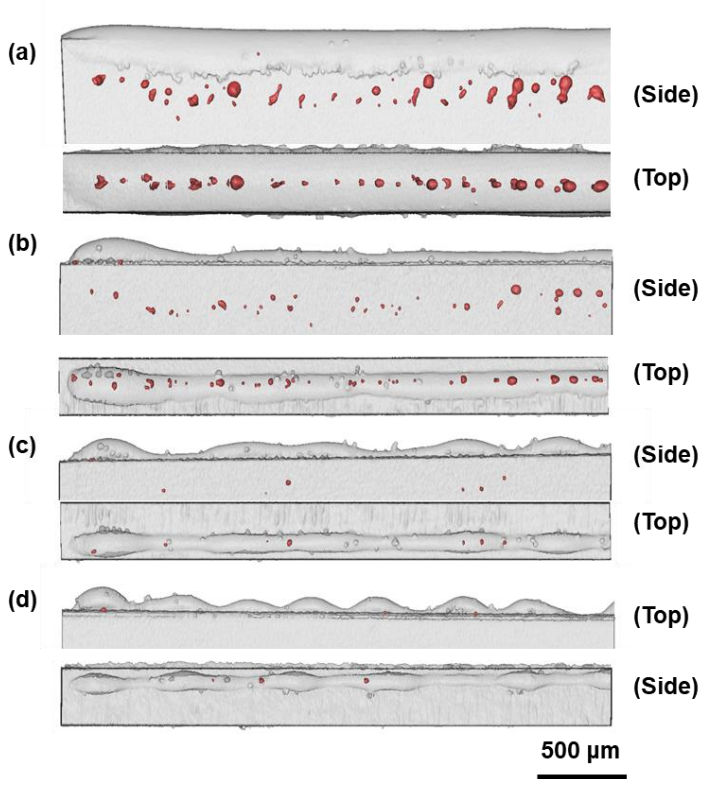

Supplementary Figure 2: XCT rendered images of 4 melt tracks at four specific energies of (a) 0.095, (b) 0.067, (c) 0.047, and (d) 0.033 MJ m^{-1} s$^{-1/2}$.

1.2. Powder characterisation

The morphology of a virgin nitrogen gas atomised Inconel 625 powder (LPW Technology Ltd., UK) was examined by a JEOL JSM-6610LV scanning electron microscope (SEM). Its particle size distribution was extracted using SEM images and image analysis techniques depicted in ref [3]. The chemical composition was characterised by energy dispersive spectroscopy (EDS) and glow discharge - optical emission spectrometry (GD-OES) according to the ASTM E1086. The sulphur content was analysed by LECO CS-400 whereas the oxygen and nitrogen contents were measured by an inert gas fusion analyser (ONH836, Leco cooperation, USA) according to the ASTM standard E1019. [4] Our results show that the Inconel 625 powder contains low oxygen and nitrogen contents, indicating that it is a fresh powder.

The SEM image (**Supplementary Figure 3**) shows that most powder particles have a spherical shape and their surface is covered with satellite particles; some powder particles exhibit open pores (see inset). Under the conditions studied, we assumed the powder morphology played no role in the molten pool and defect dynamics. The particle size distribution of the Inconel 625 powder is in the range of 5 to 70 µm with a mode, d_{10}, $d_{50,}$ and d_{90} which are 30, 30, 42, and 54 µm, respectively (**Supplementary Figure 3**). The elemental compositional analysis of Inconel 625 is summarised in **Supplementary Table 1**. The GD-OES results show that the Inconel 625 powder has low oxygen and nitrogen contents, indicating that it is a fresh batch of powder.

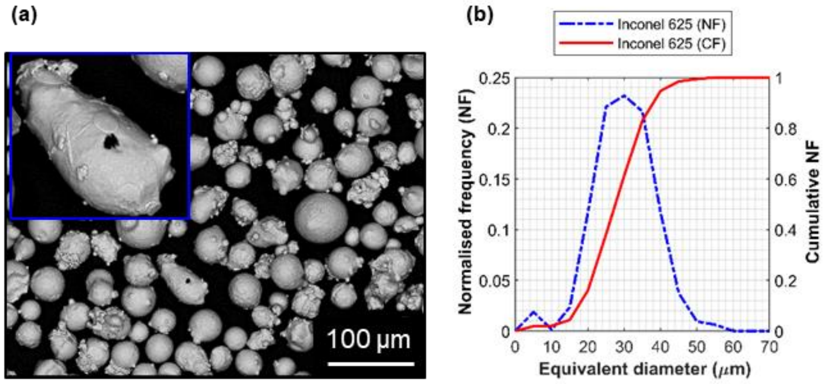

Supplementary Figure 3: Powder characterisation of Inconel 625: (a) SEM image with an inset showing an open pore on a powder particle and (b) the particle size distribution.

Supplementary Table 1:The elemental composition of Inconel 625 powder was analysed by EDS and GD-OES. The EDS results were normalised by not taking oxygen content into account.

Method	Elemental compositions of Inconel 625 (weight %)													
	Ni	Cr	Mo	Nb	Ta	Fe	Al	Ti	Co	P	S	Mn	O	N
EDS	65.5 ± 0.4	22.9 ± 0.3	6.9	3	0.3	1.3 ± 0.1	0.1 ± 0.1	-	-	-	-	-	-	-
GD-OES	64	21	9	4	0.01	2	0.1	0.1	1.1	0.01	0.002	0.02	0.02	0.01

1.3. Melt volume extraction

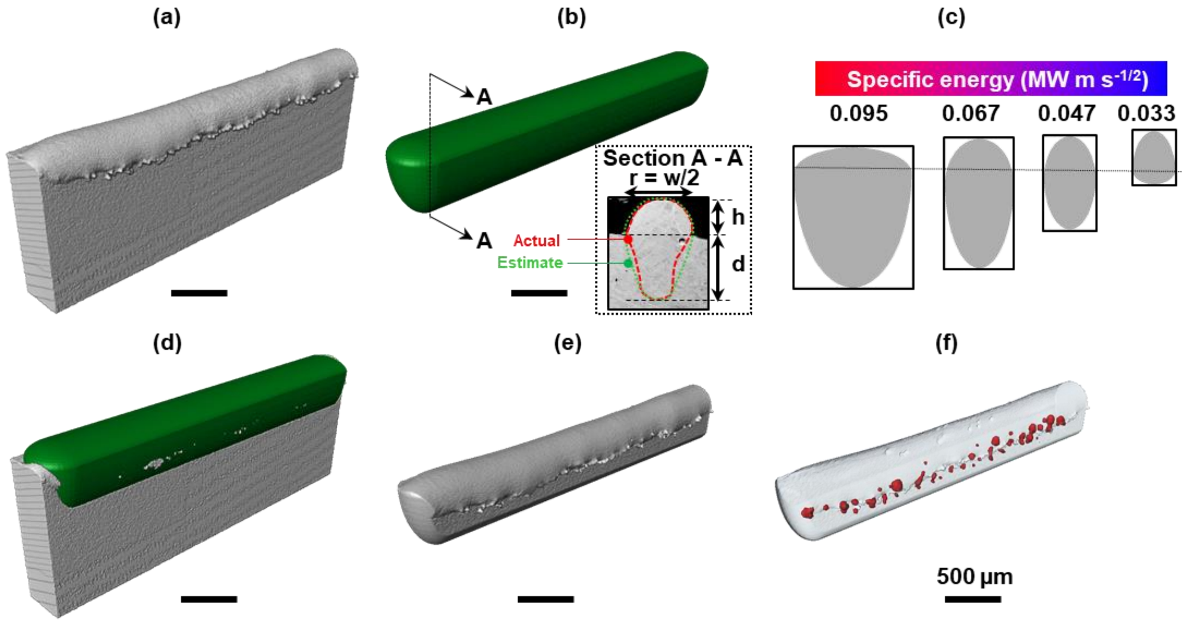

Supplementary Figure 4: Method for extracting a melt volume from XCT scan: (a) 3D rendered image of melt track on the substrate, (b) a CAD model of the melt volume is reconstructed by the maximum measured melt depth (D), height (H), and width (W) of the melt track from the SEM inset, wherein $r = 0.5 * W$. (c) The section A-A profile changes depending on the input specific energy, see measurements in **supplementary information**; (d) a rendered image shows (b) is aligned with (a), (e) lastly, the melt volume is extracted by performing $(a) * (b)$. (f) illustrates the melt volume overlays with its porosity.

Supplementary Table 2: Summary of the 3D pore analysis performed on AM Inconel 625 samples from Figure 3. D_{eq} stands for the pore volume equivalent diameter.

Scan velocity (mm s^{-1})	*Specific energy, (MJ m^{-1} s$^{-1/2}$)*	*Estimated melt volume (mm^{3})*	*Mean D_{eq} (µm)*	*Maximum D_{eq} (µm)*	*Porosity (%) = Pore vol / EMV*	*Sphericity*
50	0.95	0.41	39 ± 21	88	0.61	0.86 ± 0.10
100	0.67	0.21	29 ± 11	56	0.35	0.87 ± 0.10
200	0.47	0.11	22 ± 8	34	0.06	0.90 ± 0.05
7400	0.34	0.02	18 ± 9	32	0.16	0.88 ± 0.17

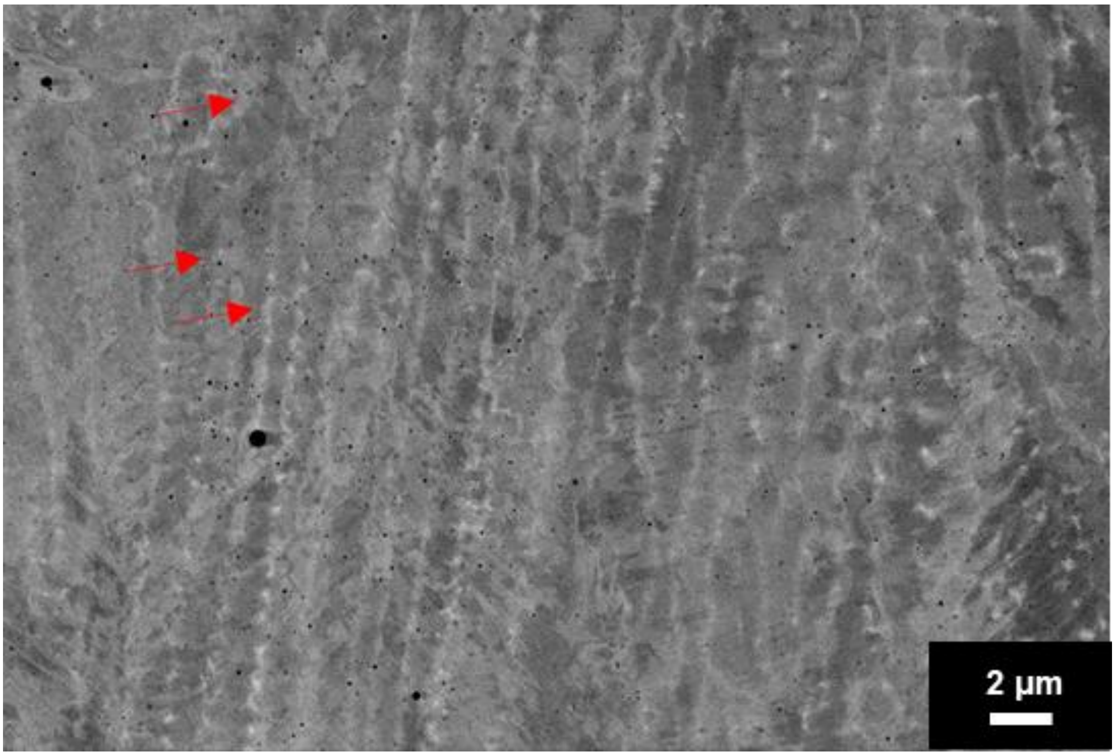

Supplementary Figure 5: SEM images show the presence of fine precipitates (black dots) in the γ matrix (see example in red arrows)

Supplementary Table 3: XCT measurements of track width (*W*), track height (*H*), melt depth (*D*), and over track length (*TL*) whereas melt pool length (*L)* is measured by the high fidelity simulation.

**Sample #**	**Specific energy (MJ m^{-1} s$^{-1/2}$)**	***W* (μm)**	**H (μm)**	***D* (μm)**	***TL* (μm)**	***L* (μm)**	***L/W ratio***	***2πW***
150 W 50 mm s^{-1}	**0.095**	434	94	421	2946	-	-	2726
150 W 100 mm s^{-1}	**0.067**	250	166	312	2894	450	1.8	1570
150 W 200 mm s^{-1}	**0.047**	190	156	188	2904	420	2.2	1193
150 W 400 mm s^{-1}	**0.033**	156	144	53	2916	380	2.4	980

1.4. Phase identification by X-ray diffraction

The Inconel 625 powder and AM tracks were examined by XRD for phase identification using a Smartlab diffractometer (Rigaku, Japan) and Profex [5]. The SmartLab was set with a 5º soller slit, two 15 mm wide receiving slits, and a 2D detector (Rigaku's HyPix 3000, Japan). The X-ray beam (45 kV and 200 mA) was set to a 200 µm spot using a collimator. The position of the X-ray source and the sample was aligned using a video camera (located inside the diffractometer) and the Smartlab guidance software (Rigaku, Japan). We calculated the lattice parameters using Bragg's law on the acquired XRD patterns. X-ray diffraction (XRD) and phase identification were also performed using a PANalytical X′Pert Pro MPD series automated spectrometer (Malvern Instruments, UK) with a Cu Kα radiation (λ = 0. 1540 nm) and 2θ angles ranging from 20° to 100°. Data analysis was carried out using open-source software - Profex [5]**.** The XRD analysis (**Supplementary figure 4**) only shows the presence of *γ* matrix in the Inconel 625 powder and AM tracks. The calculated lattice parameter, *a*, for powder and AM tracks is 3.599 Å ± a maximum scatter of 0.002 (see details in **Supplementary Table 4**) and is very similar to the reported value in ref [6].

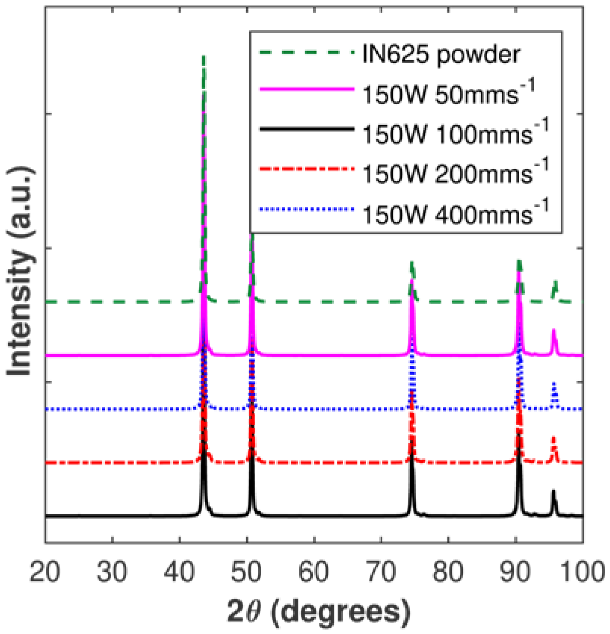

Supplementary Figure 6: X-ray diffraction (XRD) analysis on Inconel 625 powder and melt tracks (P = 150 W and v = 50 – 400 mm s^{-1})

Supplementary Table 4: Lattice parameter analysis.

**Sample #**	**Specific energy (MJ m^{-1} $s^{-1/2}$)**	**(111)**	**(200)**	**(220)**	**(311)**	**Lattice parameter, a, (Å) (mean ± scattering error)**
Powder	-	3.597	3.598	3.599	3.600	3.599 ± 0.001
150 W 50 mm s^{-1}	0.095	3.595	3.597	3.598	3.598	3.597 ± 0.001
150 W 100 mm s^{-1}	0.067	3.599	3.600	3.600	3.600	3.600 ± 0.001
150 W 200 mm s^{-1}	0.047	3.597	3.598	3.600	3.600	3.599 ± 0.002
150 W 400 mm s^{-1}	0.033	3.597	3.598	3.599	3.599	3.598 ± 0.001

Supplementary Table 5: Estimate the wavelength of the humps. *experimentally determined and **average of 0.047 and 0.033 (MJ m^{-1} $s^{-1/2}$)

**Specific energy (MJ m^{-1} $s^{-1/2}$)**	**Mean temperature (K)**	**γ (N m^{-1})**	**ρ_l (Kg m^{-3})**	**ρ_g (Kg m^{-3})**	**v_l (m/s)**	**v_g (m/s)**	**Wei's approximation λ, (mm)**	**Measured λ, (mm)**
0.067	1982.6	1.813868	7086.668	1.78	1*	2.15**	0.2	1.779
0.047	1974.9	1.814715	7093.059	1.78	1.963	2.1	6.4	0.914
0.033	1951.9	1.817245	7112.149	1.78	2	2.18	102	0.0623

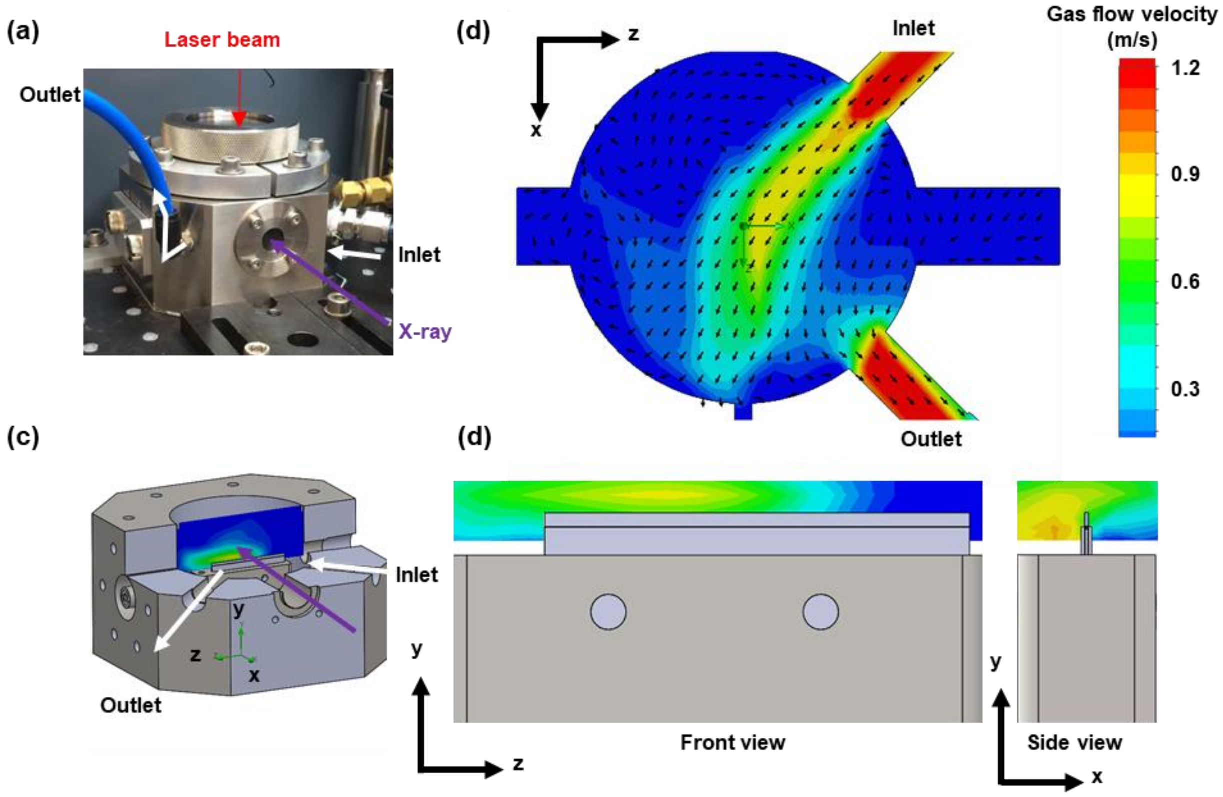

Supplementary Figure 7: Gas flow simulation inside the In situ and Operando Process Replicator (ISOPR). (a) an image of the ISOPR, (b) shows the flow vector within the sample chamber in the x-z plane wherein the inert gas flows from the inlet position to the sample area and the gas outlet, (c) shows that the gas flow profile relative to the y-z plane, and (d) a zoom-in flow profile on the y-z and x-y planes relative to the powder bed.

Supplementary Video 1: Video shows the evolution of melt track and pores during LPBF of Inconel 625 (P = 150 W, v = 50 mm s^{-1}, t = 100 µm, d = 50 µm and SE = 0.095 MJ m^{-1} $s^{-0.5}$).

Supplementary Video 2: Pore tracking video shows which pore (red outlines) has been extracted for subsequent pore area analysis during LPBF of Inconel 625 (P = 150 W, v = 50 mm s^{-1}, t = 100 µm, d = 50 µm and SE = 0.095 MJ m^{-1} $s^{-0.5}$).

Supplementary Video 3: Pore tracking video shows which pore (red outlines) has been extracted for subsequent pore area analysis during LPBF of Inconel 625 (P = 150 W, v = 100 mm s^{-1}, t = 100 µm, d = 50 µm and SE = 0.067 MJ m^{-1} $s^{-0.5}$).

2. Author contributions

CLAL, BS, and PDL conceived the project. CLAL performs SEM, 2D radiography, topological characterisation, XRD, and XCT analysis. EG performed XCT scanning. CLAL, EG, SM, and RA set up and ran the *in situ* X-ray imaging experiments. CLAL, EG, and MM prepared SEM track samples. MM performed SEM and EBSD. DL develops the multiphase and multiphysics simulation model and CLAL performs the simulation analysis. SM performs the gas flow simulation on the ISOPR. CLAL led the writing of the manuscript with all authors contributing.

3. References

[1] J.J. Valencia, P.N. Quested, ASM Handb. Cast. 15 (2008) 468–481.

[2] L. Cao, X. Yuan, Materials (Basel). 12 (2019) 2272.

[3] C.L.A. Leung, S. Marussi, R.C.. Atwood, M. Towrie, P.J.. Withers, P.D. Lee, Nat. Commun. 9 (2018) 1355.

[4] C.L.A. Leung, S. Marussi, M. Towrie, R.C. Atwood, P.J. Withers, P.D. Lee, Acta Mater. 166 (2019) 294–305.

[5] N. Doebelin, R. Kleeberg, J. Appl. Crystallogr. 48 (2015) 1573–1580.

[6] S.K. Rai, A. Kumar, V. Shankar, T. Jayakumar, K. Bhanu Sankara Rao, B. Raj, Scr. Mater. 51 (2004) 59–63.
